# Arbuscular Mycorrhizal Colonization Alters Subcellular Distribution and Chemical Forms of Cadmium in *Medicago sativa* L. and Resists Cadmium Toxicity

**DOI:** 10.1371/journal.pone.0048669

**Published:** 2012-11-06

**Authors:** Yuanpeng Wang, Jing Huang, Yanzheng Gao

**Affiliations:** 1 Institute of Organic Contaminant Control and Soil Remediation, College of Resource and Environmental Sciences, Nanjing Agricultural University, Nanjing, Jiangsu Province, P.R. China; 2 Department of Chemical and Biochemical Engineering, College of Chemistry and Chemical Engineering, Xiamen University, Xiamen, Fujian Province, P.R. China; Dowling College, United States of America

## Abstract

Some plants can tolerate and even detoxify soils contaminated with heavy metals. This detoxification ability may depend on what chemical forms of metals are taken up by plants and how the plants distribute the toxins in their tissues. This, in turn, may have an important impact on phytoremediation. We investigated the impact of arbuscular mycorrhizal (AM) fungus, *Glomus intraradices*, on the subcellular distribution and chemical forms of cadmium (Cd) in alfalfa (*Medicago sativa* L.) that were grown in Cd-added soils. The fungus significantly colonized alfalfa roots by day 25 after planting. Colonization of alfalfa by *G. intraradices* in soils contaminated with Cd ranged from 17% to 69% after 25–60 days and then decreased to 43%. The biomass of plant shoots with AM fungi showed significant 1.7-fold increases compared to no AM fungi addition under the treatment of 20 mg·kg^−1^ Cd. Concentrations of Cd in the shoots of alfalfa under 0.5, 5, and 20 mg·kg^−1^ Cd without AM fungal inoculation are 1.87, 2.92, and 2.38 times higher, respectively, than those of fungi-inoculated plants. Fungal inoculation increased Cd (37.2–80.5%) in the cell walls of roots and shoots and decreased in membranes after 80 days of incubation compared to untreated plants. The proportion of the inactive forms of Cd in roots was higher in fungi-treated plants than in controls. Furthermore, although fungi-treated plants had less overall Cd in subcellular fragments in shoots, they had more inactive Cd in shoots than did control plants. These results provide a basis for further research on plant-microbe symbioses in soils contaminated with heavy metals, which may potentially help us develop management regimes for phytoremediation.

## Introduction

Cadmium (Cd) is a widespread hazardous heavy metal. Many agricultural soils have elevated concentrations of Cd resulting from management practices such the application of sewage sludge or animal manure. Mining activities can also lead to high concentrations of Cd in surrounding lands. These and other practices may threaten environmental quality and sustainable food production [1], [2]. Excessive concentrations of Cd are toxic to plants and profoundly interfere with a series of physiological processes such as enzyme activity, respiration, photosynthesis, and nutrient assimilation [3].

However, some plants that can grow in Cd-contaminated soils have evolved mechanisms for tolerating heavy metals inside plant cells [4]. There is some evidence that metal tolerance and detoxification in plants can be achieved by confining toxins to a subcellular distribution or by changing their chemical structure. For example, zinc (Zn) and Cd are preferentially stored in vacuoles of the epidermal and mesophyll cells of the Zn/Cd hyperaccumulator *Thlaspi caerulescens* [5], [6]. Similarly, in *Brassica juncea* and *Arabidopsis thaliana*, leaf trichomes appear to be preferential storage and detoxification sites for Cd [7]. In Cd-tolerant *Salix viminalis*, pectin-rich layers of the collenchyma cell walls of leaf veins are an important Cd sink [8].

Although some recent studies have shown that several plants can tolerate and detoxify Cd, few studies have examined the effects of soil microbes that form associations with these plants, especially arbuscular mycorrhizal (AM) fungi. AM fungi play significant roles in the recycling of plant nutrients, maintenance of soil structure, detoxification of noxious chemicals, control of plant pests, and regulation of plant growth and its interactions with the soil environment [9], [10]. Plant-microbe symbioses are ubiquitous in both natural and most anthropogenically influenced soils [11]–[13]. In addition, AM fungi have been shown to transport and immobilize Cd in the root system, which reduces the detrimental effects of the metal on plant physiology [14]. Therefore, the use of mycorrhizal plants for land remediation has been proposed. However, how AM fungi might affect other aspects of heavy metal accumulation and distribution in plants remains uncertain.

**Figure 1:**
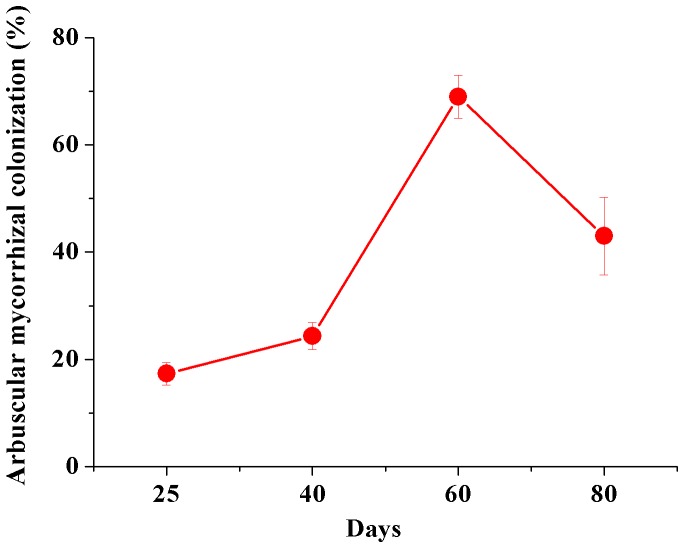
Arbuscular mycorrhizal colonization (%) of alfalfa (*Medicago sativa* L.) exposed to 20 mg kg^−1^ Cd in soil.

Alfalfa (*Medicago sativa* L.) is a deep-rooting perennial plant with high biomass production. It grows quickly, is tolerant to drought, and does not have any reported environmental hazards. In this sense, it is an ideal natural resource for the remediation of contaminated soils [15], [16]. Batch laboratory experiments have determined that alfalfa can bind various heavy metal ions [17]. In addition, alfalfa has been shown to tolerate and take up heavy metals from soil; however, relatively few studies have investigated the effects of AM fungi on the uptake and distribution of metals (and the forms of metals) in alfalfa [18], [19].

In the present study, we investigated the effects of AM fungi on the growth and subcellular uptake of Cd of alfalfa planted in soil contaminated with Cd. We also examined forms of Cd taken up by the plants and quantified the amounts of each form of Cd in plant tissues. Our results elucidate the interaction among AM fungi, alfalfa, and Cd-contaminated soil, and provide new insights into how AM fungi may affect plant uptake and distribution of heavy metals.

**Figure 2:**
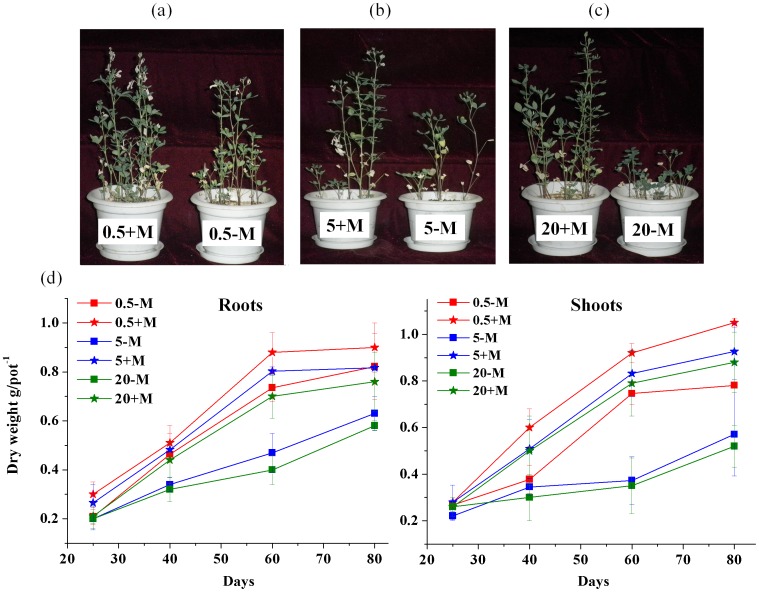
Effect of Cd^2+^ toxicity on plant growth in plants inoculated with the AM fungus after 80 days (a: 0.5 mg kg^−1^ Cd treatment; b: 5 mg kg^−1^ treatment; c: 20 mg kg^−1^ treatment) and the biomass of alfalfa for untreated plants (No AMF) and treated plants (AMF) after 25–80 days of growth in 0.5, 5 and 20 mg kg^−1^ Cd soil (d) (0.5-M means 0.5 mg kg^−1^ Cd treatment without AM fungal inoculation; 0.5+M means 0.5 mg kg^−1^ Cd treatment with AM fungal inoculation).

## Materials and Methods

### Soil Characterization

Uncontaminated loamy soil (13.4% sand, 61.9% silt, 24.7% clay, pH 6.0) was collected from the top 20 cm of a field site in Jiangning, Nanjing, China, and the total Cd concentration in soil was very low at 0.014 mg·kg^−1^. According to the United States Department of Agriculture soil taxonomy, the collected soil was Eutric Planosol [20]. The soil was air-dried and sieved (<0.45 mm) to remove plant materials, soil macrofauna, and stones. The samples were sieved using a 10-mm mesh sieve and wrapped in aluminum foil packages of approximately 1 kg. Each package was autoclaved at 121°C for 30 min to inactivate native AM fungi. A representative uncontaminated soil sample was analyzed for organic matter (2.41%), total nitrogen (1.02 g·kg^−1^), available phosphorous (7.70 mg·kg^−1^), and available potassium (60.38 mg·kg^−1^).

**Figure 3:**
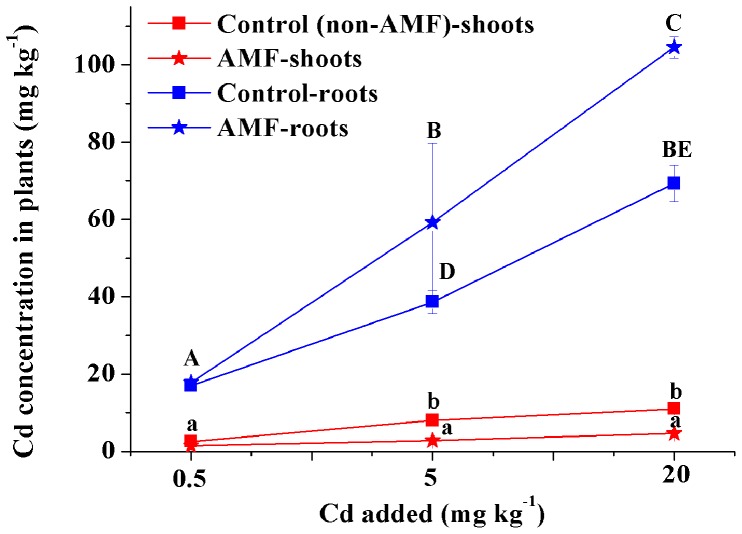
Accumulated Cd in alfalfa inoculated with *G. intraradices* after 80 days of seedling growth. Sharing a common lowercase are not significantly different in the Cd concentration in plant shoots and the same capital are not significantly different in the Cd concentration in plant roots (*P*<0.05).

### Cd Treatments

Subsamples contaminated with Cd were prepared from bulk soil. Briefly, Cd concentrations of 0.5, 5, and 20 mg·kg^−1^ soil were used, added as CdCl_2_·2.5H_2_O. The Cd salt was dissolved in distilled water, sprayed onto the soil samples, and mixed thoroughly. Then the treated soil samples were incubated at 70% maximum field water-holding capacity for 3 months. After incubation, the subsamples were air-dried naturally, fully homogenized, and stored for use. Three months pre-treatment of the soil samples may have an impact on soil heavy metals availability. However, this pre-treatment made the soil samples close to real contaminated soil. Although Cd had different speciation in the soil and the availability of Cd were not presented in the study, the total concentration of Cd is a good representation of the contamination.

**Table 1:** Subcellular distribution of Cd in alfalfa.

			The amount of heavy metals in each cell fraction per unit (kg) (mg/kg)				
Plant	Cd (mg/kg)	AM fungi	Cell wall	Soluble fraction	Organelles	Membranes	Recoveryb) (%)
Root	0.5	+Ma)	10.77±0.52b)	4.09±0.08	3.34±0.25	0.37±0.01	85.2c)
		−Md)	5.42±0.16	0.75±0.38	3.84±0.55	0.51±0.01	80.5
	5	+M	44.57±11.32	14.59±5.70	3.98±3.53	0.17±0.04	94.7
		−M	21.26±0.76	12.75±0.89	8.79±1.26	1.06±0.09	86.0
	20	+M	57.27±1.80	26.06±0.82	16.06±0.09	2.30±0.03	97.2
		−M	23.44±1.98	17.09±1.49	21.92±1.24	0.62±0.01	93.4
Shoot	0.5	+M	0.47±0.02	0.48±0.06	0.06±0.00	−	85.1
		−M	2.00±0.02	0.32±0.01	0.25±0.02	−	90.2
	5	+M	0.91±0.03	0.94±0.01	0.22±0.03	0.38±0.02	91.9
		−M	5.24±0.01	0.63±0.31	0.76±0.01	0.47±0.01	87.3
	20	+M	2.02±0.01	0.97±0.01	0.20±0.00	0.51±0.01	87.3
		−M	7.64±0.61	0.54±0.01	0.73±0.01	0.58±0.01	90.6

a) With AM fungal inoculation.

b) Mean±standard deviation(n = 3).

c) Percentage recovery (%) = (cell wall +organelle+ soluble fraction+ membrane)×100%/total.

d) Without AM fungal inoculation.

### Seedling Preparation and Incubation

Seeds of alfalfa were soaked in distilled water for 30 min and then germinated in commercial potting mixture. Seedlings were watered daily with a nutrient solution for 2 weeks. Then uniform seedlings were selected and transplanted to porcelain pots, each containing 0.4 kg Cd-contaminated soil. *Glomus intraradices* was selected as the AM fungi, based on our previous study [21]. Mycorrhizal pots were inoculated with 15 g *G. intraradices* spores. Non-mycorrhizal controls received an equivalent amount of sterilized inoculum to provide similar conditions, except for the absence of the active mycorrhizal fungi. All pots were equilibrated in a glass greenhouse to 50% water-holding capacity. All treatments were carried out in triplicate.

**Figure 4:**
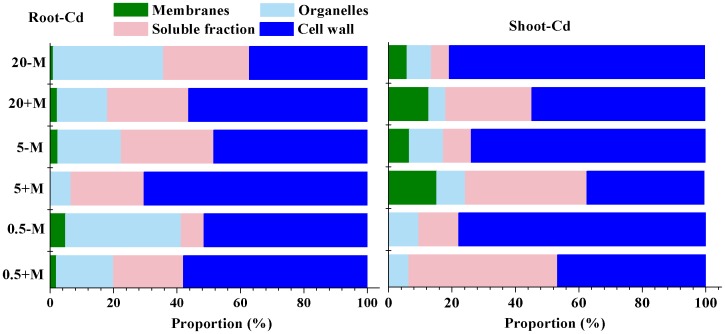
The proportion of Cd with a subcellular distribution in alfalfa.

The plants were grown in the greenhouse with average day and night temperatures of 32°C and 16°C, respectively. For plant biomass and colonization by the AM fungi *G. intraradices,* the plants were harvested at different time (25, 40, 60 and 80 days after transplantation). For subcellular and chemical forms of Cd, the plants were harvested after 80 days after transplantation. They were removed from the pots, and their roots were washed in tap water to remove soil and then washed with deionized water.

### Mycorrhizal Analysis

Root samples of the alfalfa plants were cleared and stained as described by Phillips and Hayman [22], and then a 1-g subsample of fresh root was randomly taken and cut into approximately 1 cm pieces to estimate the proportion of total root length colonized by the AM fungi. Mycorrhizal colonization was determined using the gridline intersect method [23]. Briefly, the stained root segments were arranged lengthwise on a thin layer of PVA mounted on a microscope slide. A hairline graticule inserted into the eyepiece of a compound microscope acted as a line of intersection with the roots. Fungal structures at each intersection were calculated by observation at 200× magnification (Nikon, TE2000).

**Table 2:** Distribution of each chemical form of Cd in alfalfa.

			The amount of Cd in each fraction per unit (kg) (mg/kg)					
Plant	Cd (mg/kg)	AM fungi	F_ethanol_	Fd-H_2_O	F_Nacl_	F_HAc_	F_HCl_	F_residue_
Root	0.5	+M	2.91±0.06	4.15±0.04	6.73±0.03	6.09±0.06	2.58±0.05	1.50±0.08
		−M	2.57±0.04	4.59±0.06	5.57±0.06	4.12±0.42	2.07±0.05	1.06±0.06
	5	+M	8.20±1.78	8.99±1.99	14.79±3.17	13.80±3.01	7.19±1.58	3.06±0.66
		−M	7.24±0.09	8.07±0.05	14.32±0.33	10.11±0.04	4.52±0.04	1.38±0.04
	20	+M	14.6±0.28	19.41±0.27	32.62±0.26	29.80±0.02	12.15±0.39	8.44±0.01
		−M	8.11±0.02	13.03±0.25	23.84±0.18	16.93±0.19	5.98±0.03	3.58±0.04
Shoot	0.5	+M	0.32±0.00	2.86±1.26	1.21±0.01	0.22±0.01	0.30±0.00	1.04±0.03
		−M	0.64±0.01	2.35±0.02	3.85±0.00	0.07±0.00	0.58±0.02	1.30±0.04
	5	+M	0.61±0.04	0.99±0.27	2.25±0.02	0.38±0.02	0.61±0.05	2.10±0.08
		−M	1.84±0.01	6.79±0.12	0.89±0.39	0.90±0.39	1.33±0.00	3.65±0.00
	20	+M	1.07±0.00	2.37±0.01	4.33±0.01	0.70±0.01	0.81±0.00	3.95±0.01
		−M	1.07±0.01	6.40±0.03	15.50±0.02	0.27±0.02	1.40±0.01	3.27±0.00

### Subcellular Fractions

Frozen materials were homogenized in cold extract buffer containing 50 mM Hanks’ balanced salt solution (HEPES), 500 mM sucrose, 1.0 mM dithiothreitol (DTT), 5.0 mM ascorbic acid, and 1.0% (w/v) polyvinyl polypyrrolidone (PVPP), and adjusted to pH 7.5 with NaOH. Cells were separated into the four fractions of cell wall, soluble fraction, organelle, and cell membranes using the differential centrifugation technique reported by Lozano-Rodriguez et al. [24] with some modifications [4]. The homogenate was sieved through a nylon cloth (100 µm mesh size) and washed with extraction buffer. This residue, together with the pellet retained after centrifugation of the filtrate at 100 g for 5 min, constituted the cell wall-bound metals. The supernatant contained the remaining metals, which were further separated into three fractions. First, the supernatant was centrifuged at 10,000 g for 30 min, producing a pellet containing the organelles. Then it was centrifuged at 100,000 g for 30 min, resulting in a pellet containing the cell membranes. The remaining supernatant contained the soluble fraction. All steps were performed at 4°C.

**Figure 5:**
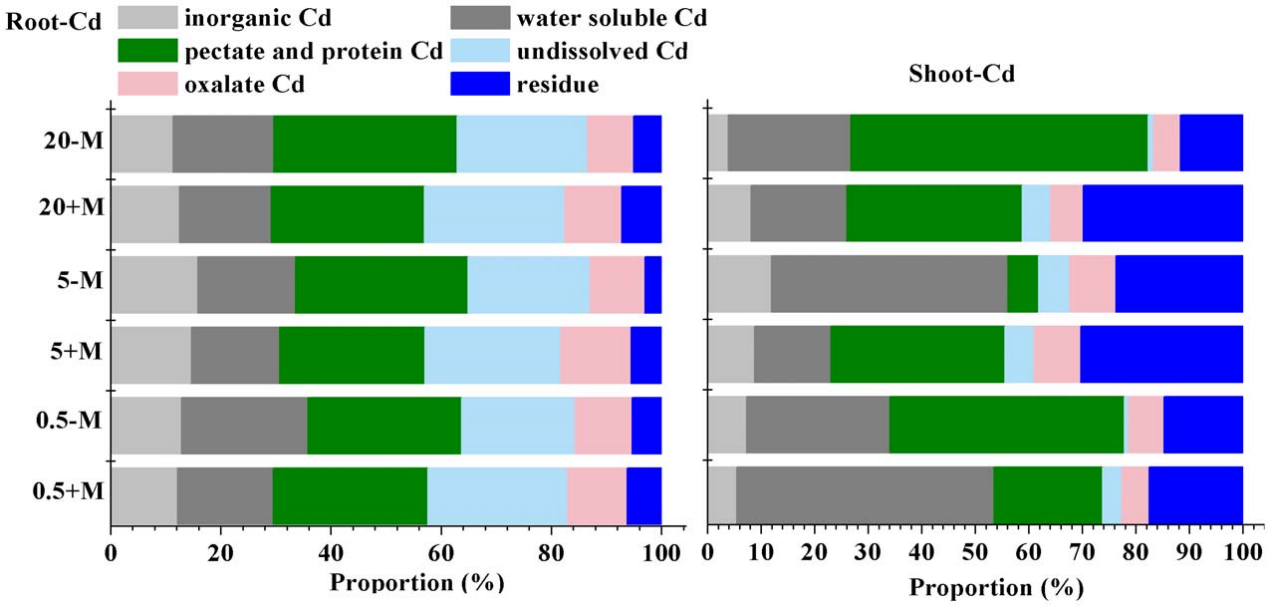
The proportion of each chemical form of Cd in alfalfa.

### Chemical Forms

To determine the chemical forms of Cd in alfalfa, the following extractions were performed in the following order: (1) 80% ethanol, extracting inorganic Cd giving priority to nitrate/nitrite, chloride, and aminophenol cadmium; (2) deionized water (d-H_2_O), extracting water soluble Cd-organic acid complexes and Cd(H_2_PO_4_)_2_; (3) 1 M NaCl, extracting pectates and protein-integrated Cd; (4) 2% acetic acid (HAc), extracting undissolved cadmium phosphate including CdHPO_4_ and Cd_3_(PO_4_)_2_ and other Cd-phosphate complexes; and (5) 0.6 M HCl, extracting cadmium oxalate [4].

Frozen tissues were homogenized in extraction solution with a mortar and pestle, diluted at a ratio of 1∶100 (w/v), and shaken for 22 h at 25°C. Then the homogenate was centrifuged at 5000×g for 10 min, obtaining the first supernatant solution in a conical beaker. The sedimentation was re-suspended twice in extraction solution, shaken for 2 h at 25°C, and centrifuged at 5000×g for 10 min. Then the supernatants of the three suspensions and centrifuge steps for each of the five extraction solutions were pooled. Each pooled solution was evaporated on an electric plate at 70°C to constant weight.

### Analysis of Heavy Metals

Before metal analysis, 5 µL concentrated HNO_3_ was added to each 1 mL sample solution. Both plant materials and the centrifuged fractions were dried at 105°C for 24 h. Plant materials were weighed to determine the dry weight (DW). Then all plant materials and centrifuged fractions were wet-digested in concentrated HNO_3_:HClO_4_ (7∶3, v:v). Metal concentrations of all plant materials and all centrifuged fractions were determined using inductively coupled plasma optical emission spectrometry (ICP-OES, Optima 2000DV, Perkin-Elmer Co., USA).

### Statistical Analysis

All data were analyzed using Microsoft Excel, Origin and SPSS. The treatment effects were carried out with one-way ANOVA and the LSD multiple range test was used to determine the statistical significance (*P*<0.05) between pairs with SPSS.

## Results

### Colonization by the AM Fungi *G. intraradices*

Because the soils in our greenhouse experiments were sterilized, there was no colonization by the AM fungi or any indigenous fungi in untreated plants. In plants inoculated with *G. intraradices*, mycelia in endodermis of root cells were observed after 25 days of growth. They first penetrated root cells, and then slowly branched from the outer cortical cells to the inner cortical cells. Some of intracellular hyphae were straight and slowly began to form arbuscule. Vesicles could be detected at 60 days after inoculation. At 80 days, spores formed and filled the entire cell (Fig. S1). Colonization of alfalfa by *G. intraradices* in soils contaminated with Cd ranged from 17% to 69% after 25–60 days and then decreased to 43% (Fig. 1).

### Plant Growth

The biomass of all plants increased with incubation time, reaching maximum values at 80 days. The shoots and roots biomass of untreated plants decreased with increasing Cd concentration, however, AM fungal inoculation significantly increased the biomass of roots and shoots compared to untreated plants. When Cd addition concentration was 20 mg kg^−1^, the biomass of plant shoots with AM fungi showed significant 1.7-fold increases compared to no AM fungal addition (*P*<0.05). On the other hand, there were no significant differences on the biomass with AM fungal inoculation under 0.5 mg kg^−1^ and 5 mg kg^−1^ Cd treatments (Fig. 2).

### Plant Uptake of Cd

Concentrations of Cd in the shoots of alfalfa were 2.56, 8.02, and 11.02 mg·kg^−1^ under 0.5, 5, and 20 mg·kg^−1^ Cd without AM fungal inoculation; these values are 1.87, 2.92, and 2.38 times higher, respectively, than those of fungi-inoculated plants. However, Cd concentrations were higher in the roots than in the shoots, especially in fungi-inoculated plants (Fig. 3).

### Subcellular Distribution of Cd

Table 1 summarizes the subcellular distribution of Cd in shoots and roots. The recovery rate of Cd uptake was more than 85% for most treatments, and Cd concentrations in subcellular fractions increased with increasing heavy metal concentrations with or without AM inoculation. Most Cd was present in the soluble and cell wall fractions, with very little in the membrane.

Fungi-inoculated plants had less Cd in membranes after 80 days of incubation compared to untreated plants. In addition, fungal inoculation led to less Cd accumulation in subcellular fractions of shoots, increased levels (37.2–80.5%) in the cell walls of roots and shoots (Fig. 4), and decreased levels in organelles and membranes compared to controls.

### Chemical Forms of Cd

Cd of different forms increased with increasing Cd exposure (Table 2). In roots, undissolved and pectates and protein-integrated Cd were predominant in both untreated and fungi-inoculated plants, whereas other forms were rare. In shoots, pectates and protein-integrated Cd and water soluble Cd, and Cd residues, made up the largest proportion of Cd, followed by inorganic and oxalate Cd.

The undissolved and oxalate Cd, and Cd residues, generally showed no or little toxicity to plants. Hence, these three forms were considered to be inactive. The proportion of these inactive forms of Cd in roots was higher in fungi-treated plants than in controls. Furthermore, although fungi-treated plants had less overall Cd in subcellular fragments in shoots, they had more inactive Cd in shoots than did control plants (Fig. 5).

## Discussion

Certain plants can be used to clean up contaminated soils and waters. This detoxification ability may depend on what chemical forms of metals are taken up by plants and how the plants distribute the toxins in their tissues, and hence these factors may impact phytoremediation efforts [25]–[27]. One such plant is alfalfa, which is widely distributed in both contaminated and uncontaminated areas of China [15]. We investigated the subcellular distribution and chemical forms of Cd in alfalfa (*Medicago sativa* L.) inoculated with the arbuscular mycorrhizal (AM) fungi *Glomus intraradices* and planted in contaminated soil. Our results indicate that AM fungi have the potential ability of reducing Cd accumulation and beneficial consequences of mycorrhizal associations on plant growth in heavy metals contaminated soil.

High concentrations of Cd were toxic to alfalfa plants lacking AM fungi, which significantly decreased biomass. However, AM fungal inoculation significantly increased the biomass of roots and shoots compared to untreated plants and there were no significant differences on the biomass with AM fungal inoculation under 0.5 mg kg^−1^ and 5 mg kg^−1^ Cd treatments (Fig. 2). In present study, AM fungi alter Cd transformation in alfalfa and resist Cd toxicity by reducing Cd uptake from roots to shoots, distributing Cd in the subcellular fractions of cell walls, restricting Cd accumulation in membranes, and isolating Cd in an inactive state in roots and shoots.

Plants that accumulate and/or tolerate Cd have evolved mechanisms to resist taking up metals in the soil and/or to tolerate metals inside cells [28]. Some mycorrhizal fungi that grow in association with plants have been shown to reduce the accumulation of certain heavy metals in the shoots of host plants, presumably by increasing metal retention within roots [11], [29]. Indeed, in the present study, plants inoculated with AM fungi had larger amounts of Cd in their roots and smaller amounts in their shoots (Fig. 3), improving the plants’ resistance to Cd toxicity compared to controls. In addition, AM-treated plants distributed more Cd in subcellular fractions of tissues, further reducing Cd accumulation in membranes (Table 1, Fig 4). Excessive accumulation in plant membranes would destroy cell activity and thus inhibit plant growth [25], [26]. Hence, selective distribution of toxins might be an important strategy for heavy metal tolerance and detoxification in plants.

The fungi-treated plants also sequestered more Cd in cell walls. Cell walls are mainly composed of cellulose, hemicellulose, pectin, and protein, whose surfaces tend to be negatively charged [30], serving as efficient sites for sequestering toxins.

Heavy metals that crossed cell walls eventually dissolved and became incorporated into organelles. However, we found that plants with AM fungal inoculation accumulated more Cd in the soluble fractions of roots and shoots. That is, more Cd remained in solution and never entered organelles, suggesting that the soluble fraction serves as another storage compartment for toxins in both roots and shoots. Therefore, the cell matrix between cells and organelles could be considered an intracellular buffer [31]. Soluble cellular components not only store heavy metals but also contain organo-ligands, which are mainly sulfur-rich peptides, organic alkali, and organic acids. Complexation of metals with organo-ligands within such storage sites decreases free ion activity and thus reduces toxicity [32].

Finally, AM fungi also improved tolerance to Cd by seemingly converting Cd into inactive forms, as treated plants had significantly more inactive forms of Cd in roots and shoots than did untreated plants. Different chemical species of heavy metals have different biological functions, with distinct toxicities and migration patterns [4], [33]. For example, water-soluble Cd has a high capacity to migrate and is more deleterious to plant cells, whereas Cd in forms such as metal-phosphate complexes shows no or little toxicity to plants. In the present study, there was a significant increase in the proportion of inactive forms of Cd in the subcellular fractions of both roots and shoots of fungi-treated plants compared to controls (Fig. 5). Among these fractions, a large proportion of Cd was integrated with pectates and protein, suggesting that Cd may have been chelated by a polar material such as a hydroxyl or carboxyl group, forming a non-toxic complex.

Although heavy metal pollutants had significant effects on AM fungi in soil [34], [35], numerous studies have demonstrated that symbiotic interactions between plants and AM fungi may improve plant tolerance to and uptake of heavy metals [36]–[38]. So inoculation of AM fungi would be important in phytoremediation of soils contaminated with heavy metals. Even some propagules of indigenous fungi already exist in soil, there may be competition among AM fungi with different ability of colonization. Therefore, inoculation of target AM fungal species that have been demonstrated to be effective in detoxification would be helpful to improve the efficiency of phytoremediation.

In summary, inoculation of AM fungi significantly enhanced the growth of alfalfa plants in a Cd-contaminated soil, increased total Cd in roots but decreased Cd concentrations in shoots. Our results also showed that AM fungi increased the proportion of Cd in cell wall while reducing the proportion in organelles and membranes. Moreover, AM fungi increased the proportion of inactive forms of Cd in roots and shoots. Together, our results illustrate that AM fungi enhanced plant resistance or tolerance to Cd through altering both the forms of Cd and the distribution of Cd among different plant tissues. These findings provide new insights into the roles of plant-microbe symbioses in mediating the impact of heavy metals on plants and highlight the need of further research on the contribution of AMF to phytoremediation.

## Supporting Information

Figure S1 **The status of roots inoculated with AM fungus** ***G. intraradices*** **. (a) 25 days, (b) 40 days, (c) 60 days (d) 80days.**
(DOC)
