# Supplementary material for: Arbuscular Mycorrhizal Colonization Alters Subcellular Distribution and Chemical Forms of Cadmium in Medicago sativa L. and Resists Cadmium Toxicity
Source: PLoS One. 2012 Nov 6;7(11):e48669. doi: 10.1371/journal.pone.0048669 (PMC3490862; doi:10.1371/journal.pone.0048669)
Supplement: Figure S1 — The status of roots inoculated with AM fungus G. intraradices . (a) 25 days, (b) 40 days, (c) 60 days (d) 80days. (DOC) [file pone.0048669.s001.doc]

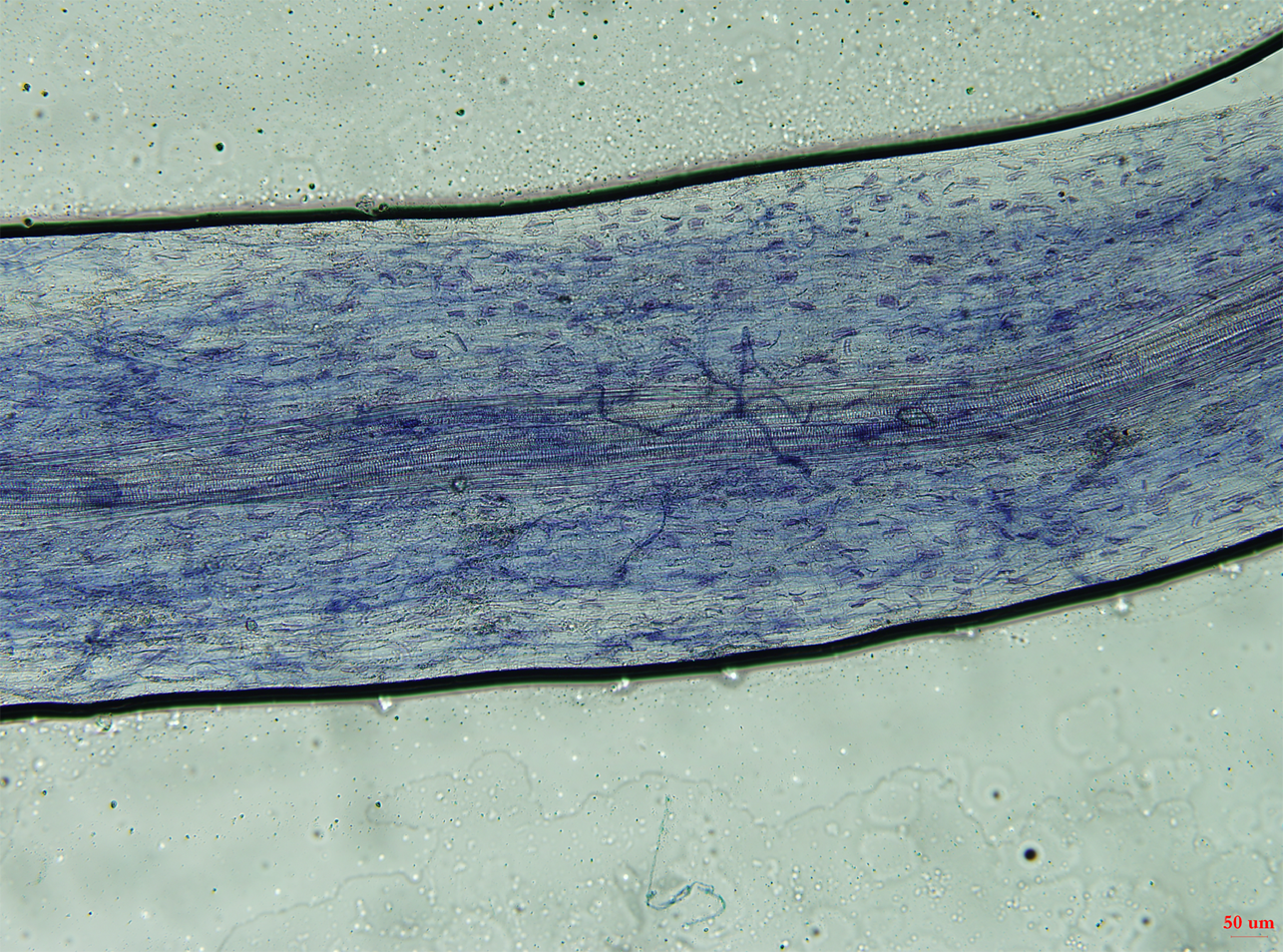

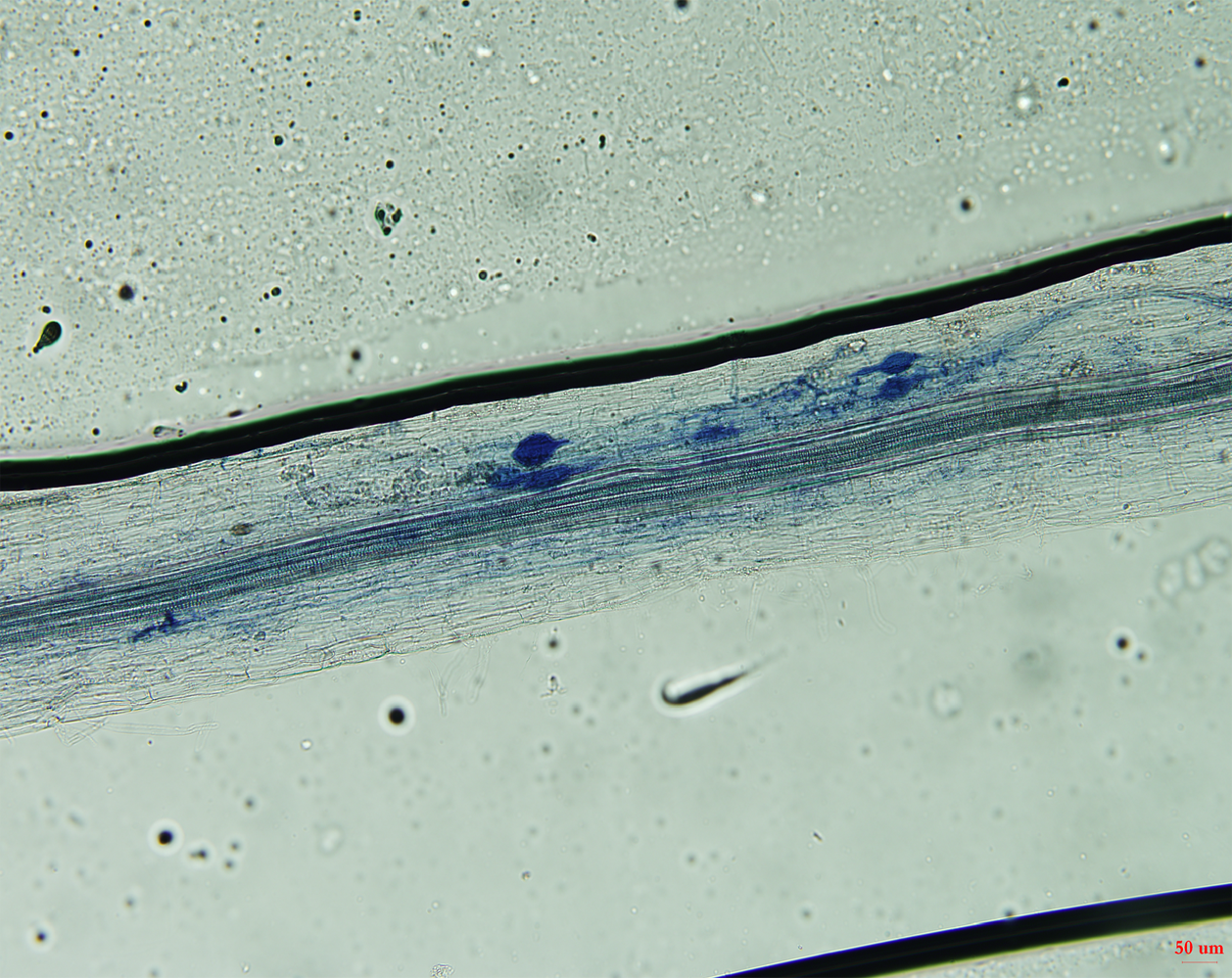


(b)

(a)


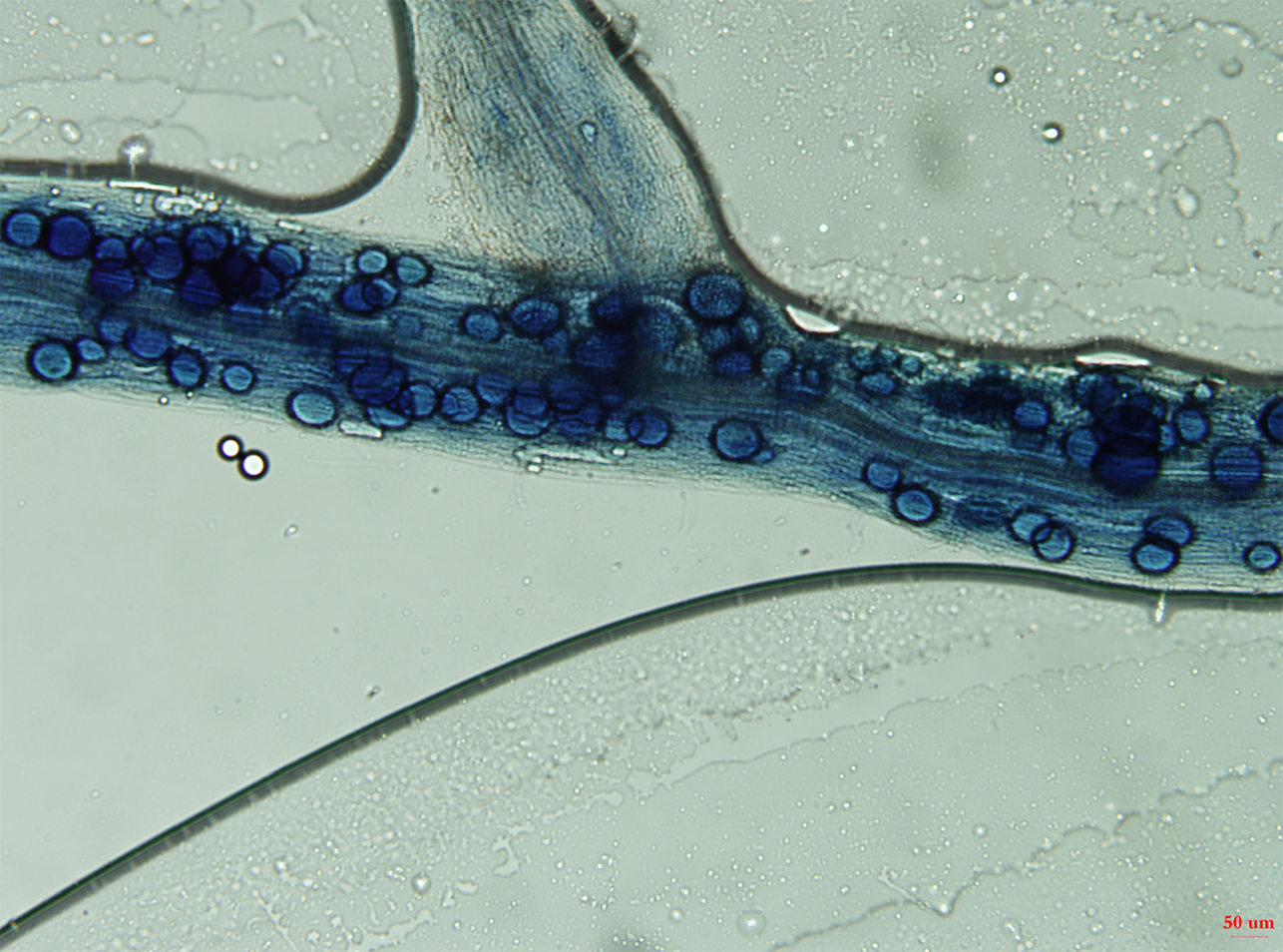


(c)


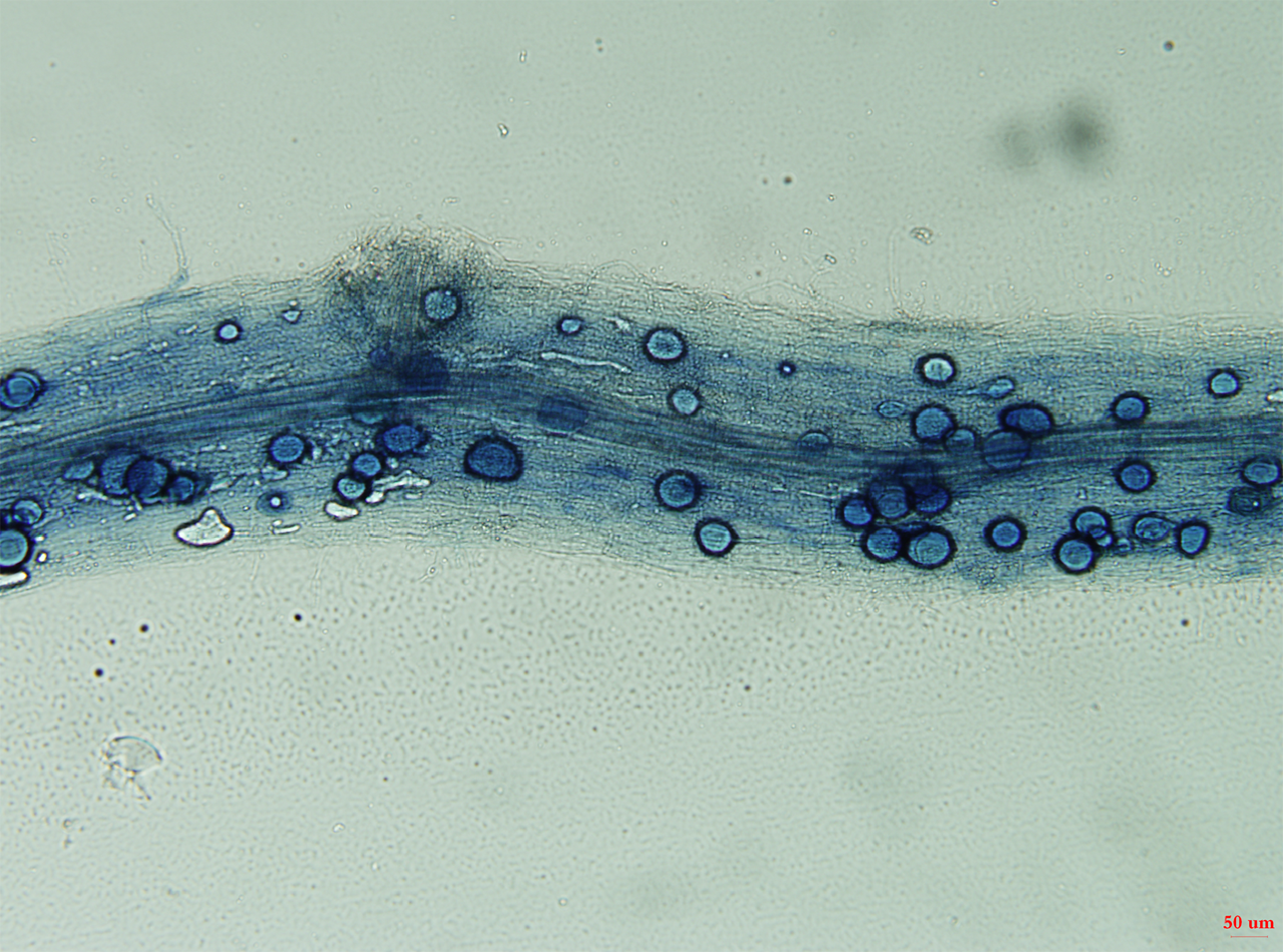


(d)

Figure S1. The status of roots inoculated with AM fungus *G. intraradices*. (a) 25 days, (b) 40 days, (c) 60 days (d) 80days.
